# Metabolic markers associated with insulin resistance predict type 2 diabetes in Koreans with normal blood pressure or prehypertension

**DOI:** 10.1186/s12933-016-0368-7

**Published:** 2016-03-22

**Authors:** Ki-Chul Sung, Hyun-Young Park, Min-Ju Kim, Gerald Reaven

**Affiliations:** Division of Cardiology, Department of Medicine, Kangbuk Samsung Hospital, Sungkyunkwan University School of Medicine, #108, Pyung Dong, Jongro-Ku, Seoul, 110-746 Republic of Korea; Division of Cardiovascular and Rare Diseases, Center for Biomedical Science, Korea National Institute of Health, 187 Osongsaengmyeng 2-ro, Osong-eup, Heungdeok-gu, Cheongju, Chungbuk 361-951 Republic of Korea; Division of Cardiovascular Medicine, Stanford University School of Medicine, Stanford, CA 94305 USA

**Keywords:** Type 2 diabetes insulin resistance TG/HDL-C ratio prehypertension

## Abstract

**Background:**

Questions remain as to the association between essential hypertension and increased incidence of type 2 diabetes (T2DM). The premise of this analysis is that insulin resistance/compensatory hyperinsulinemia is a major predictor of T2DM, and the greater the prevalence of insulin resistance within any population, normotensive or hypertensive, the more likely T2DM will develop. The hypothesis to be tested is that surrogate estimates of insulin resistance will predict incident T2DM to a significant degree in persons with normal blood pressure or prehypertension.

**Methods:**

Analysis of data from a population-based survey of 10, 038 inhabitants of rural and urban areas of Korea, ≥40 years-old, initiated in 2001, with measures of demographic and metabolic characteristics at baseline and 8-years later. Participants were classified as having normal blood pressure or prehypertension, and three simple manifestations of insulin resistance related to the pathophysiology of T2DM used to predict incident T2DM: (1) glycemia (plasma glucose concentration 2-hour after 75 g oral glucose challenge = 2-hour PG); (2) hyperinsulinemia (plasma insulin concentration 2-hour after 75 g oral glucose challenge = 2-hour PI); and (3) dyslipidemia (ratio of fasting plasma triglyceride/high/density lipoprotein cholesterol concentration = TG/HDL-C ratio).

**Results:**

Fully adjusted hazard ratios (HR, 95 % CI) for incident T2DM were highest (P < 0.001) in the quartile of individuals with the highest 2-hour PG concentrations, ranging from 5.84 (3.37–10.1) in women with prehypertension to 12.2 (7.12–21.00) in men with normal blood pressure. T2DM also developed to a significantly greater degree in subjects within the highest quartile of TG/HDL-C ratios, with HRs varying from 2.91 (1.63–2.58) in women with prehypertension (P < 0.001) to 1.77 (1.12–2.81, P < 0.05) in men with prehypertension. The least predictive index of insulin resistance was the 2-hour PI concentration. Subjects with normal blood pressure in the highest quartile of 2-hour PI concentrations were significantly associated with incident T2DM, with HRs of 1.5 (1.02–2.20, P = 0.25) and 2.02 (1.35–3.02, P < 0.001), in men and women, respectively. Finally, incidence of T2DM in the highest quartile was somewhat greater in patients with prehypertension, irrespective of predictor.

**Conclusions:**

Metabolic variables associated with insulin resistance (glycemia, insulinemia, and dyslipidemia) predict the development of T2DM in patients with either normal blood pressure or prehypertension.

**Electronic supplementary material:**

The online version of this article (doi:10.1186/s12933-016-0368-7) contains supplementary material, which is available to authorized users.

## Background

In a recent publication, Emdin and colleagues pointed-out that despite biological rationale for a relationship between elevated blood pressure and incident type 2 diabetes (T2DM), 12 of 30 cohort studies reviewed could not identify evidence of this association. Furthermore, the remaining 18 studies “reported a considerably variable strength of association [1]”. In an effort to obtain a more definitive view of the putative association between elevated blood pressure and T2DM, they analyzed medical records of 4.1 million individuals, free of hypertension and T2DM, in a U.K. primary care setting, as well as performing a meta-analysis of existing prospective studies. The results of their analysis documented a significant association between elevations of blood pressure and T2DM, and concluded that “further investigation is needed to determine whether this association is causal.”

The association between elevated blood pressure and T2DM is not limited to inhabitants of the U.K., and results of the recent Korean Genome and Epidemiological Study have demonstrated that this relationship also exists in patients with prehypertension [2]. Assuming the presence of an association between elevations in blood pressure and T2DM, it remains to be seen, as pointed out by Emdin, et al. [1], if the two abnormalities are causally related. The overall hypothesis underlying this analysis is that the association between hypertension and T2DM is causal in nature, and related to the role of insulin resistance as a major risk factor in the genesis of both hypertension and T2DM [3–5]. Put most simply, insulin resistance is a predictor of T2DM [3, 4], and the greater the prevalence of this defect in a population [5], the more at risk of T2DM they will be. The more specific hypothesis underlying this analysis is that surrogate estimates of insulin resistance will predict incident T2DM not only in a normal population, but also in patients with prehypertension.

## Methods

### Study participants

**Fig. 1:**
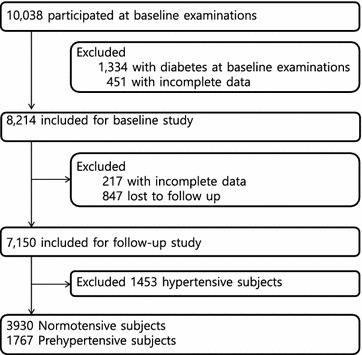
Study flow chart showing subjects screened, included and followed up

The Korean Genome and Epidemiology Study [2], a population-based prospective cohort study, was initiated to investigate prevalence in Korea of risk factors for chronic disease, as well as incident disease. The survey began in 2001–2002, included 10,038 participants ≥40 years of age, and follow-up examinations were performed every 2 years. Specimens have been collected from residents in both rural (Anseong) and urban (Ansan) areas. Baseline and 8-year follow-up data were obtained from the Center for Genome Science in the National Institute of Health, Korea. Details of the present cohort have been described elsewhere [6]. Of the initial cohort, complete data were available on 5697 participants classified at baseline as having normal blood pressure (n = 3930) or prehypertension (n = 1767), and analysis of these data form the substance of this report (Fig. 1). The study protocol was approved by the Institutional Review Board of the Korea Centers for Disease Control and Prevention, and written informed consent was obtained from all participants.

### Demographic and metabolic measurements

Waist circumference (WC) was measured at the midpoint between the ribs and the iliac crest in the standing position. Body weight and height were measured to the nearest 0.1 kg and 0.1 cm, with body mass index (BMI) calculated as weight (kg) divided by height (m^2^). BP was measured after a 5 min rest period in the supine position, with measurements taken at least twice at 30-s intervals and averaged. Blood samples were collected after at least an 8 h fast. Plasma glucose (PG), total cholesterol, high-density lipoprotein cholesterol (HDL-C), and triglyceride (TG) concentrations were measured enzymatically, and low-density lipoprotein cholesterol concentrations estimated by the Friedwald formula [7] Plasma insulin (PI) concentrations were measured by radioimmunoassay and hemoglobin A1C (HbA1C) concentrations by high-performance liquid chromatography.

### Definition of diabetes mellitus and hypertension

Diabetes mellitus was diagnosed according to criteria of the American Diabetes Association as either a fasting PG ≥126 mg/dL, a PG concentration ≥200 mg/dL 2-hours after an oral glucose challenge, an HbA1C ≥6.5 %, or use of an oral hypoglycemic agent [8]. Participants were classified according to the Seventh Report of the Joint National Committee Prevention, Detection, Evaluation, and Treatment of High Blood Pressure [9] as having normal blood pressure (<120 mm Hg systolic and <80 mm Hg diastolic) or prehypertension (120–139 mm Hg systolic or 80–89 mm Hg diastolic). The 1453 participants considered to have hypertension at baseline, based upon an elevated blood pressure (≥40 mm Hg systolic or ≥90 mm Hg diastolic) or use of blood pressure-lowering medication, were excluded from further analysis to avoid the possible adverse effects of hypertension-related peripheral vascular disease on insulin resistance as contributing to their 21 % incidence of T2DM.

### Surrogate estimates of insulin resistance

The ability of three different manifestations of insulin resistance to identify enhanced risk of T2DM in participants with either normal BP or prehypertension at baseline was evaluated.

1. 2-hour PG: PG concentration 2-hours after an oral glucose challenge to estimate the degree to which the overall glycemic status has decompensated in the face of a decrease in insulin action.
2. 2-hour PI: PI concentration 2-hours after an oral glucose challenge to estimate compensatory insulin response in the face of insulin resistance [10, 11].
3. Fasting plasma TG/HDL-C ratio: lipid factors associated with insulin resistance, known to be significantly correlated with insulin resistance and adverse clinical outcome [12–15].

### Statistical analysis

Distribution testing for normality was performed using the Shapiro–Wilk test, with the data log-transformed to obtain normalized distributions. The baseline characteristics of subjects were expressed as mean ± S.D., or geometric means with 95 % confidence intervals (CIs). Differences between groups were compared by one-way analysis of variance for continuous variables and χ2 tests for categorical variables. The geometric means of log-transformed variables were back-transformed for ease of interpretation and reported with their 95 % CIs. Diabetes incidence rate was calculated per 1000 person-years for 2-hour PG, 2-hour PI, and plasma TG/HDL-C concentration ratio. Cox proportional hazards models were used to analyze time at risk and the association, HOMA-IR, 2-hour insulin, and TG/HDL-C ratio, and reported as hazard ratios (HRs) and 95 % CIs. Participants with 1st quartile of each variable at baseline were considered the reference group. Values of P < 0.05 were considered statistically significant. All data were analyzed using SPSS software (version 21.0; SPSS, Chicago, IL, USA).

## Results

Baseline demographic and metabolic characteristics in the total experimental population are presented in Table 1. In addition, Table 1 contains comparison of these experimental variables in the normal and prehypertension subgroups. These data indicate that patients with prehypertension were older, with higher values for body mass index and waist circumference, and higher blood pressures. With the exception of the 2-hour plasma insulin, high-density lipoprotein cholesterol, and low-density lipoprotein concentration, the values were all other metabolic variables were higher in those with prehypertension.

**Table 1:** **Comparison of baseline characteristics among participants with normal blood pressure and prehypertension**

Variable	All (n = 5697)	Normal BP (n = 3930)	Prehypertension (n = 1767)	P value
Age (year)	50.6 ± 8.5	49.3 ± 7.9	53.6 ± 8.9	<0.001
BMI (kg/m2)	24.1 ± 3.0	23.9 ± 2.9	24.6 ± 3.1	<0.001
WC (cm)	81.1 ± 8.4	80.0 ± 8.2	83.6 ± 8.5	<0.001
SBP (mmHg)	110.4 ± 12.5	104.3 ± 9.0	124.1 ± 7.2	<0.001
DBP (mmHg)	71.3 ± 9.1	67.5 ± 7.7	79.8 ± 5.6	<0.001
FPG (mg/dL)	82.4 ± 8.3	81.9 ± 8.1	83.5 ± 8.7	<0.001
2-hour PG (mg/dL)	112.0 ± 29.5	111.2 ± 28.9	113.7 ± 30.9	0.003
FPI (μIU/mL)	7.34 ± 4.83	7.18 ± 4.67	7.71 ± 5.15	<0.001
2-hour PI (μU/mL)	26.4 ± 24.6	26.2 ± 24.0	26.8 ± 25.9	0.354
HOMA-IR	1.50 ± 1.01	1.46 ± 0.97	1.59 ± 1.10	<0.001
Hemoglobin A1C (%)	5.52 ± 0.34	5.50 ± 0.33	5.57 ± 0.35	<0.001
TC (mg/dL)	188.0 ± 33.2	186.3 ± 32.4	191.7 ± 34.8	<0.001
HDL-C (mg/dL)	45.3 ± 9.9	45.3 ± 9.7	45.4 ± 10.2	0.845
LDL-C (mg/dL)	114.5 ± 30.6	114.1 ± 29.6	115.5 ± 32.7	0.105
TG (mg/dL)	140.6 ± 64.6	134.5 ± 61.5	154.1 ± 69.0	<0.001
TG/HDL-C	3.38 ± 2.04	3.23 ± 1.95	3.70 ± 2.19	<0.001

The data are expressed as mean ± standard deviation. Statistical differences between groups were compared with one-way ANOVA

*BMI* body mass index, *WC* waist circumference, *SBP* systolic blood pressure, *DBP* diastolic blood pressure, *FPG* fasting plasma glucose, *2*-*hour PG* plasma glucose 2-hours post-glucose challenge, *FPI* fasting insulin, *2*-*hour PI* plasma insulin 2-hours post-glucose challenge, *HOMA*-*IR* homeostatic model for insulin resistance, *TC* total cholesterol, *HDL*-*C* high-density-lipoprotein cholesterol, *LDL*-*C* low-density lipoprotein cholesterol, *TG* triglyceride

Table 2 presents the sex-stratified risk for incident T2DM in normal subjects and patients with prehypertension divided into quartiles of the on the basis of their 2-hour PG concentration. Incident T2DM was greatest in the highest quartile of those with prehypertension in both men (43 vs. 31 %) and women (37 vs. 25 %). In general, the higher the quartile, the greater the fully adjusted HR, varying in quartile 4 from 5.84 (3.37–10.1) in prehypertensive women to 12.2 (7.12–21.0) in men with normal blood pressure (all P < 0.001).

**Table 2:** Sex-stratified risk for incident diabetes by quartile of 2-hour plasma glucose among participants with normal blood pressure and prehypertension

	Number at risk	Diabetes cases	Unadjusted		Model 1		Model 2	
			HR (95 % CI)	P for trend	HR (95 % CI)	P for trend	HR (95 % CI)	P for trend
Normal BP								
Men								
Quartile 1	462	15	1 (reference)	<0.001	1 (reference)	<0.001	1 (reference)	<0.001
Quartile 2	428	29	2.08 (1.12–3.88)*		2.08 (1.12–3.88)*		2.07 (1.11–3.86)*	
Quartile 3	439	45	3.20 (1.78–5.74)**		3.20 (1.78–5.73)**		3.28 (1.82–5.90)**	
Quartile 4	443	138	11.8 (6.95–20.2)**		11.8 (6.94–20.2)**		12.2 (7.12–21.0)**	
Women								
Quartile 1	555	18	1 (reference)	<0.001	1 (reference)	<0.001	1 (reference)	<0.001
Quartile 2	562	23	1.32 (0.71–2.45)		1.30 (0.70–2.40)		1.27 (0.68–2.35)	
Quartile 3	521	41	2.52 (1.45–4.39)*		2.45 (1.41–4.27)*		2.24 (1.28–3.91)*	
Quartile 4	517	130	9.04 (5.52–14.8)**		8.76 (5.35–14.4)**		8.10 (4.92–13.3)**	
Prehypertension								
Men								
Quartile 1	238	12	1 (reference)	<0.001	1 (reference)	<0.001	1 (reference)	<0.001
Quartile 2	246	21	1.66 (0.82–3.38)		1.68 (0.83–3.42)		1.60 (0.79–3.26)	
Quartile 3	235	36	3.26 (1.70–6.27)**		3.21 (1.67–6.17)**		3.09 (1.60–5.95)*	
Quartile 4	230	99	10.4 (5.72–19.0)**		10.5 (5.75–19.1)**		9.96 (5.44–18.2)**	
Women								
Quartile 1	213	16	1 (reference)	<0.001	1 (reference)	<0.001	1 (reference)	<0.001
Quartile 2	197	10	0.71 (0.32–1.57)		0.72 (0.33–1.58)		0.70 (0.32–1.54)	
Quartile 3	209	28	1.84 (0.99–3.40)		1.83 (0.99–3.39)		1.78 (0.96–3.30)	
Quartile 4	199	73	6.16 (3.58–10.6)**		6.13 (3.56–10.5)**		5.84 (3.37–10.1)**	

Model 1: adjusted for age

Model 2: adjusted for the variables in model 1 and body mass index, family history of diabetes (yes or no), education (less than high school, high school or equivalent, or college or above), alcohol use (current or non-current), and smoking status (current or non-current)

* P < 0.05, ** *P* < 0.001

Table 3 contains a similar comparison, but in this case the quartiles were created as a function of the magnitude of the TG/HDL-C ratio. Incident T2DM was again greatest in the highest quartile of those with prehypertension in both men (24 vs. 18 %) and women (24 vs. 16 %). When compared to the 2-hour PG, the actual values of the HRs were lower when the TG/HDL-C ratio was used to predict incident T2DM. However, the HRs in the upper quartile and incident T2DM remained statistically significantly associated in the fully adjusted model in both experimental groups and in men and women.

**Table 3:** Sex-stratified risk for incident diabetes by quartile of TG/HDL-C among participants with normal blood pressure and prehypertension

	Number at risk	Diabetes cases	Unadjusted		Model 1		Model 2	
			HR (95 % CI)	P for trend	HR (95 % CI)	P for trend	HR (95 % CI)	P for trend
Normal BP								
Men								
Quartile 1	443	39	1 (reference)	<0.001	1 (reference)	<0.001	1 (reference)	<0.001
Quartile 2	444	47	1.15 (0.75-1.76)		1.16 (0.76-1.77)		1.18 (0.77-1.81)	
Quartile 3	444	61	1.53 (1.02-2.28)*		1.54 (1.03-2.30)*		1.54 (1.02-2.32)*	
Quartile 4	443	82	2.16 (1.47-3.16)**		2.21 (1.51-3.23)**		2.08 (1.39-3.12)**	
Women								
Quartile 1	539	32	1 (reference)	<0.001	1 (reference)	<0.001	1 (reference)	<0.001
Quartile 2	539	44	1.43 (0.91–2.27)		1.41 (0.89-2.23)		1.55 (0.98-2.46)	
Quartile 3	539	49	1.60 (1.02–2.51)*		1.53 (0.98-2.41)		1.63 (1.03-2.57)*	
Quartile 4	539	88	3.00 (1.97–4.47)**		2.72 (1.80-4.12)*		2.58 (1.70-3.94)**	
Prehypertension								
Men								
Quartile 1	237	37	1 (reference)	<0.001	1 (reference)	<0.001	1 (reference)	<0.001
Quartile 2	237	37	0.92 (0.58–1.45)		0.93 (0.59–1.46)		0.96 (0.60–1.54)	
Quartile 3	238	37	0.98 (0.62–1.55)		1.01 (0.64–1.59)		1.03 (0.63–1.68)	
Quartile 4	237	57	1.54 (1.02–2.33)*		1.65 (1.09–2.50)*		1.77 (1.12–2.81)*	
Women								
Quartile 1	206	16	1 (reference)	<0.001	1 (reference)	<0.001	1 (reference)	<0.001
Quartile 2	203	22	1.39 (0.73–2.65)		1.39 (0.73–2.64)		1.35 (0.70–2.59)	
Quartile 3	205	41	2.78 (1.56 –4.96)*		2.76 (1.55–4.92)*		2.66 (1.47–4.79)*	
Quartile 4	204	48	3.23 (1.83–5.69)**		3.18 (1.80–5.62)**		2.91 (1.63–5.18)**	

Model 1: adjusted for age

Model 2: adjusted for the variables in model 1 and fasting plasma glucose, body mass index, family history of diabetes (yes or no), education (less than high school, high school or equivalent, or college or above), alcohol use (current or non-current), and smoking status (current or non-current)

* *P* < 0.05, ** *P* < 0.001

Table 4 depicts the sex-stratified risk for incident T2DM when persons with normal blood pressure and patients with prehypertension were stratified into quartiles on the basis of their 2-hour PI concentration. As before, incident T2DM occurred more commonly in men (26 vs. 18 %) and women (20 vs. 15 %) with prehypertension. Although this manifestation of insulin resistance predictor was the least powerful in identifying incident T2DM, the fully adjusted model still detected a significant trend (P < 0.05) between magnitude of 2-hour PI and incident T2DM in both populations and n men and women. Furthermore, the HR of the quartile with the highest 2-hour PI concentration (quartile 4) was still significantly associated to the development of T2DM in men (1.50, 1.02–2.21) and women (2.02, 1.35–3.02) with normal blood pressure.

**Table 4:** Sex-stratified risk for incident diabetes by quartile of 2 h plasma insulin among participants with normal blood pressure and prehypertension

	Number at risk	Diabetes cases	Unadjusted		Model 1		Model 2	
			HR (95 % CI)	P for trend	HR (95 % CI)	P for trend	HR (95 % CI)	P for trend
Normal BP								
Men								
Quartile 1	446	43	1 (reference)	<0.001	1 (reference)	<0.001	1 (reference)	<0.001
Quartile 2	443	48	1.06 (0.70–1.60)		1.07 (0.71–1.61)		1.09 (0.72–1.64)	
Quartile 3	440	57	1.31 (0.88–1.94)		1.34 (0.90–1.99)		1.27 (0.85–1.90)	
Quartile 4	443	79	1.90 (1.31–2.76)*		1.94 (1.34–2.82)*		1.50 (1.02–2.21)*	
Women								
Quartile 1	541	36	1 (reference)	<0.001	1 (reference)	<0.001	1 (reference)	<0.001
Quartile 2	542	41	1.13 (0.72–1.76)		1.14 (0.73–1.79)		1.16 (0.74–1.82)	
Quartile 3	543	56	1.53 (1.01–2.34)*		1.60 (1.05–2.44)*		1.60 (1.05–2.45)*	
Quartile 4	538	79	2.15 (1.45–3.18)**		2.19 (1.48–3.25)**		2.02 (1.35–3.02)**	
Prehypertension								
Men								
Quartile 1	241	35	1 (reference)	<0.001	1 (reference)	<0.001	1 (reference)	<0.001
Quartile 2	234	34	0.91 (0.57–1.46)		0.89 (0.56–1.43)		0.85 (0.53–1.37)	
Quartile 3	239	39	1.14 (0.72–1.80)		1.14 (0.72–1.80)		1.10 (0.69–1.74)	
Quartile 4	235	60	1.70 (1.12–2.58)*		1.78 (1.17–2.71)*		1.42 (0.92–2.18)	
Women								
Quartile 1	205	24	1 (reference)	<0.001	1 (reference)	<0.001	1 (reference)	<0.001
Quartile 2	204	28	1.16 (0.67–2.00)		1.15 (0.67–1.99)		1.18 (0.68–2.05)	
Quartile 3	207	33	1.26 (0.75–2.13)		1.25 (0.74–2.12)		1.38 (0.81–2.34)	
Quartile 4	201	41	1.67 (1.01–2.77)*		1.70 (1.02–2.82)*		1.63 (0.97–2.73)	

Model 1: adjusted for age

Model 2: adjusted for the variables in model 1 and fasting plasma glucose, body mass index, family history of diabetes (yes or no), education (less than high school, high school or equivalent, or college or above), alcohol use (current or non-current), and smoking status (current or non-current)

* *P* < 0.05, ** *P* < 0.001

Quartiles of all variables, baseline characteristics according to diabetes status at follow-up and risk for incident diabetes by quartile of HOMA-IR are shown in Additional file 1.

## Discussion

This analysis was based on the premise that the greater the prevalence of insulin resistance within any diagnostic category, the more likely the incident rate of T2DM will increase. In other words, the fundamental question related to the likelihood of developing T2DM is not whether a person has a normal blood pressure or prehypertension, but whether or not they are insulin resistant. Before discussing the findings that support this hypothesis, it seems crucial to address the methods used to evaluate the association between insulin resistance and T2DM in the two experimental groups. Insulin resistance in nondiabetic persons is associated with increased glycemia, hyperinsulinemia, and dyslipidemia, irrespective of whether they have normal blood pressure [16] or prehypertension [17]. Many other metabolic markers have been used to identify insulin resistance in nondiabetic persons, employing more sophisticated approaches [18–22]. For example, HOMA-IR [18] is commonly used in population-based studies to provide a surrogate estimate of insulin resistance. However, HOMA-IR is a calculated value that combines within mathematical formula measurements of PI and PG concentrations. Since the 2-hour PG was being used in the analysis as one of the variables associated with insulin resistance, we thought it prudent not to use HOMA-IR which also includes a measurement of PG. Faced with the myriad associations between insulin resistance and multiple metabolic markers [18–22], it was decided to use the simplest available measurements of the three cardinal manifestations of insulin resistance in nondiabetic individuals: glycemia (2-hour PG), hyperinsulinemia (2-hour PI), and dyslipidemia (TG/HDL-C ratio).

Within the potential limitations of the markers selected for this analysis, the results in Tables 2, 3, 4 provide experimental support for the hypothesis that the more insulin resistant an individual, whether they had normal blood pressure or prehypertension, the greater their risk of developing T2DM. Specifically, the greater was the magnitude of the manifestations of insulin resistance, whether it is glycemia, hyperinsulinemia, or dyslipidemia, the more likely the development of T2DM. Furthermore, these general findings obtained irrespective of sex and clinical diagnosis.

Although the findings in Tables 2, 3, 4 are comparable in that manifestations of insulin resistance were significantly associated with incident T2DM in both experimental populations, it should be noted that metabolic characteristics of insulin resistance were accentuated in patients with prehypertension [Table 1]. By selection, blood pressure was also elevated in those with prehypertension. These considerations bring into focus the question of causality raised by Emdin and colleagues [1]: does elevated blood pressure, per se, increase risk of T2DM, or do abnormalities that increase risk of hypertension also increase risk of T2DM? This question cannot be answered by the current data, but a strong biological argument can be made in support of the second alternative. For example, normotensive, first-degree relatives of patients with high blood pressure are insulin resistant when compared to normotensive individuals without a family history of hypertension [23–25], and surrogate markers of insulin resistance predict incident hypertension [26, 27]. Finally, there is considerable evidence that resistance to insulin-mediated glucose uptake is increased in patients with essential hypertension when compared to appropriate control groups [28–30]. On the other hand, there are important differences in the relationship between insulin resistance and T2DM as compared to its relationship to hypertension [5]. Thus, the overwhelming majority of patients with T2DM are insulin resistant, whereas only approximately 50 % of patients with essential hypertension, treated or untreated, are insulin resistant [31], and these differences in prevalence may help explain why it has been so difficult to even establish the existence of a relationship between elevated both pressure and T2DM.

There are limitations to our study that should be discussed. Firstly, we conducted a post hoc analysis of epidemiological data collected for other purposes. In addition, it is possible that our findings in inhabitants of Korea may not apply to other racial/ethnic populations. Thirdly, direct quantification of insulin resistance was not available, and our analysis was based on use of differences in dysglycemia, hyperinsulinemia, and dyslipidemia, three metabolic abnormalities characteristic of insulin resistance, to evaluate the association between insulin resistance and incident T2DM. Perhaps the most appropriate way view our findings is as hypothesis-generating; leaving it to future studies to validate, or discard, the formulation that the increased incidence of T2DM in patients with essential hypertension is related to the increased prevalence of insulin resistance in this clinical syndrome.

## Conclusion

These data demonstrate that the subset of individuals with the greatest degree of insulin resistance, whether they have normal blood pressure or prehypertension, is at increased risk to develop T2DM. As such, they are consistent with the suggestion that it is the increased prevalence of insulin resistance in patients with essential hypertension that accounts for their increased risk of T2DM. Put more simply, since prevalence of insulin resistance is increased in patients with essential hypertension [5, 28–31], and insulin resistance is a powerful predictor of T2DM [3, 4], it should not be surprising, as reported by Emdin, et al. [1], that patients with essential hypertension are at increased risk to develop T2DM. What the current results do is provide evidence that the same phenomenon seems to also be true of patients with prehypertension.

## Additional file

10.1186/s12933-016-0368-7 Quartiles of all variables, baseline characteristics according to diabetes status at follow-up and risk for incident diabetes by quartile of HOMA-IR.
