# Supplementary material for: Metabolic markers associated with insulin resistance predict type 2 diabetes in Koreans with normal blood pressure or prehypertension
Source: Cardiovasc Diabetol. 2016 Mar 22;15:47. doi: 10.1186/s12933-016-0368-7 (PMC4802716; doi:10.1186/s12933-016-0368-7)
Supplement: Supplementary file 1 — 10.1186/s12933-016-0368-7 Quartiles of all variables, baseline characteristics according to diabetes status at follow-up and risk for incident diabetes by quartile of HOMA-IR. [file 12933_2016_368_MOESM1_ESM.docx]

| **Quartile** | | **Men** | **Women** |
| --- | --- | --- | --- |
| **Normal BP** | **Insulin** | Q1: ≤4.600  Q2: ≤6.100  Q3: ≤8.100  Q4: >8.100 | Q1: ≤5.400  Q2: ≤7.000  Q3: ≤9.500  Q4: >9.500 |
|  | **HOMA-IR** | Q1: ≤0.9318  Q2: ≤1.2527  Q3: ≤1.6904  Q4: >1.6904 | Q1: ≤1.0400  Q2: ≤1.3844  Q3: ≤1.8791  Q4: >1.8791 |
|  | **TG/HDL-C** | Q1: ≤2.1024  Q2: ≤3.0800  Q3: ≤4.5583  Q4: >4.5583 | Q1: ≤1.7511  Q2: ≤2.4176  Q3: ≤3.5197  Q4: >3.5197 |
|  | **PP2 insulin** | Q1: ≤8.200  Q2: ≤16.400  Q3: ≤29.075  Q4: >29.075 | Q1: ≤11.300  Q2: ≤23.100  Q3: ≤39.900  Q4: >39.900 |
|  | **2-hour PG** | Q1: ≤87.00  Q2: ≤103.00  Q3: ≤125.75  Q4: >125.75 | Q1: ≤95.00  Q2: ≤112.00  Q3: ≤131.00  Q4: >131.00 |
| **Prehypertension** | **Insulin** | Q1: ≤4.700  Q2: ≤6.400  Q3: ≤9.000  Q4: >9.000 | Q1: ≤5.700  Q2: ≤7.500  Q3: ≤10.200  Q4: >10.200 |
|  | **HOMA-IR** | Q1: ≤0.9643  Q2: ≤1.3333  Q3: ≤1.9100  Q4: >1.9100 | Q1: ≤1.1370  Q2: ≤1.4988  Q3: ≤2.0611  Q4: >2.0611 |
|  | **TG/HDL-C** | Q1: ≤2.2586  Q2: ≤3.3488  Q3: ≤5.0780  Q4: >5.0780 | Q1: ≤2.0000  Q2: ≤2.8658  Q3: ≤4.2368  Q4: >4.2368 |
|  | **PP2 insulin** | Q1: ≤8.100  Q2: ≤16.500  Q3: ≤29.900  Q4: >29.900 | Q1: ≤11.200  Q2: ≤23.600  Q3: ≤42.100  Q4: >42.100 |
|  | **2-hour PG** | Q1: ≤87.00  Q2: ≤108.00  Q3: ≤131.00  Q4: >131.00 | Q1: ≤98.00  Q2: ≤115.00  Q3: ≤136.00  Q4: >136.00 |

Supplementary table 1. Quartiles of all variables and their ranges.

Supplementary table2**.** Comparison of Baseline Characteristics among Participants with Normal Blood Pressure According to Diabetes Status at follow-up

| **Variable** | **Men (n = 1174)** | | | | | | ***P*-value** | **Women (n = 2156)** | | | | | | ***P*-value** |
| --- | --- | --- | --- | --- | --- | --- | --- | --- | --- | --- | --- | --- | --- | --- |
|  | **No diabetes**  **(n = 1545)** | | | **Incident diabetes**  **(n = 229)** | | |  | **No diabetes**  **(n = 1943)** | | | **Incident diabetes**  **(n = 213)** | | |  |
| Age (year) | 49.7 | ± | 8.1 | 50.1 | ± | 7.8 | 0.471 | 48.9 | ± | 7.7 | 50.6 | ± | 8.2 | 0.095 |
| BMI (kg/m^2^) | 23.6 | ± | 2.7 | 24.2 | ± | 2.9 | 0.102 | 24.0 | ± | 2.9 | 25.0 | ± | 3.2 | 0.136 |
| WC (cm) | 81.5 | ± | 7.0 | 83.1 | ± | 7.4 | 0.352 | 78.4 | ± | 8.7 | 80.5 | ± | 8.8 | 0.876 |
| SBP (mmHg) | 105.1 | ± | 8.7 | 105.6 | ± | 9.1 | 0.932 | 103.4 | ± | 9.2 | 105.4 | ± | 9.2 | 0.849 |
| DBP (mmHg) | 68.7 | ± | 7.7 | 69.0 | ± | 7.4 | 0.564 | 66.5 | ± | 7.6 | 66.9 | ± | 8.6 | 0.078 |
| FPG (mg/dL) | 82.8 | ± | 8.2 | 89.2 | ± | 10.0 | <0.001 | 80.0 | ± | 6.9 | 84.1 | ± | 9.1 | <0.001 |
| 2-hour PG (mg/dL) | 103.3 | ± | 27.9 | 135.7 | ± | 33.9 | <0.001 | 111.5 | ± | 25.0 | 140.0 | ± | 29.9 | <0.001 |
| FPI (μIU/mL) | 6.64 | ± | 4.57 | 6.87 | ± | 5.24 | 0.369 | 7.51 | ± | 4.53 | 8.41 | ± | 5.46 | 0.009 |
| 2-hour PI (μU/mL) | 21.7 | ± | 20.9 | 26.4 | ± | 22.0 | 0.055 | 28.3 | ± | 23.9 | 39.7 | ± | 36.3 | <0.001 |
| HOMA-IR | 1.37 | ± | 0.95 | 1.54 | ± | 1.25 | 0.038 | 1.49 | ± | 0.91 | 1.76 | ± | 1.23 | <0.001 |
| Hemoglobin A1C (%) | 5.49 | ± | 0.32 | 5.69 | ± | 0.37 | 0.001 | 5.5 | ± | 0.32 | 5.7 | ± | 0.36 | 0.008 |
| TC (mg/dL) | 188.3 | ± | 33.0 | 192.8 | ± | 32.5 | 0.683 | 183.4 | ± | 31.7 | 190.8 | ± | 31.6 | 0.744 |
| HDL-C (mg/dL) | 44.0 | ± | 9.5 | 42.3 | ± | 9.0 | 0.542 | 46.9 | ± | 9.8 | 44.4 | ± | 9.3 | 0.473 |
| LDL-C (mg/dL) | 115.7 | ± | 30.9 | 117.0 | ± | 30.1 | 0.236 | 112.1 | ± | 28.2 | 116.9 | ± | 30.4 | 0.074 |
| TG (mg/dL) | 143.4 | ± | 65.3 | 167.6 | ± | 73.8 | 0.004 | 122.0 | ± | 52.9 | 147.9 | ± | 65.8 | <0.001 |
| TG/HDL-C | 3.54 | ± | 2.07 | 4.28 | ± | 2.41 | 0.001 | 2.82 | ± | 1.64 | 3.62 | ± | 2.17 | <0.001 |

The data are expressed as mean ± standard deviation. Statistical differences between groups were compared with independent sample t-test.

Abbreviations: BMI=body mass index; WC= waist circumference; SBP= systolic blood pressure; DBP= diastolic blood pressure; FPG= fasting plasma glucose; 2-hour PG=plasma glucose 2-hours post-glucose challenge; FPI=fasting insulin; 2-hour PI= plasma insulin 2-hours post-glucose challenge;

HOMA-IR= homeostatic model for insulin resistance; TC=total cholesterol; HDL-C= high-density-lipoprotein cholesterol; LDL-C=low-density lipoprotein cholesterol; TG=triglyceride.

Supplementary table3. Comparison of Baseline Characteristics among Participants with Prehypertension According to Diabetes Status at Follow-up

| **Variable** | **Men (n = 949)** | | | | | | ***P*-value** | **Women (n = 818)** | | | | | | ***P*-value** |
| --- | --- | --- | --- | --- | --- | --- | --- | --- | --- | --- | --- | --- | --- | --- |
|  | **No diabetes**  **(n = 781)** | | | **Incident diabetes**  **(n = 168)** | | |  | **No diabetes**  **(n = 691)** | | | **Incident diabetes**  **(n = 127)** | | |  |
| Age (year) | 52.3 | ± | 9.0 | 53.7 | ± | 8.7 | 0.063 | 54.7 | ± | 8.8 | 55.2 | ± | 8.5 | 0.559 |
| BMI (kg/m^2^) | 24.1 | ± | 2.9 | 24.8 | ± | 3.1 | 0.004 | 24.9 | ± | 3.3 | 25.8 | ± | 2.7 | 0.007 |
| WC (cm) | 83.4 | ± | 7.7 | 85.5 | ± | 7.6 | 0.001 | 83.0 | ± | 9.4 | 85.5 | ± | 8.6 | 0.004 |
| SBP (mmHg) | 122.7 | ± | 7.2 | 123.9 | ± | 7.4 | 0.042 | 125.3 | ± | 6.9 | 126.0 | ± | 6.7 | 0.344 |
| DBP (mmHg) | 80.3 | ± | 5.3 | 80.9 | ± | 6.0 | 0.205 | 78.9 | ± | 5.7 | 79.3 | ± | 5.9 | 0.517 |
| FPG (mg/dL) | 83.9 | ± | 8.5 | 89.9 | ± | 10.5 | <0.001 | 81.1 | ± | 7.2 | 84.8 | ± | 10.0 | <0.001 |
| 2-hour PG (mg/dL) | 104.3 | ± | 29.0 | 137.7 | ± | 32.7 | <0.001 | 113.7 | ± | 26.0 | 140.2 | ± | 31.7 | <0.001 |
| FPI (μIU/mL) | 7.1 | ± | 4.4 | 7.0 | ± | 3.6 | 0.716 | 8.5 | ± | 6.3 | 8.0 | ± | 3.6 | 0.349 |
| 2-hour PI (μIU/mL) | 22.8 | ± | 25.3 | 29.0 | ± | 26.3 | 0.006 | 29.6 | ± | 26.2 | 33.7 | ± | 24.9 | 0.102 |
| HOMA-IR | 1.5 | ± | 0.92 | 1.6 | ± | 0.85 | 0.327 | 1.7 | ± | 1.3 | 1.7 | ± | 0.82 | 0.814 |
| Hemoglobin A1C (%) | 5.5 | ± | 0.31 | 5.8 | ± | 0.34 | <0.001 | 5.5 | ± | 0.36 | 5.8 | ± | 0.33 | <0.001 |
| TC (mg/dL) | 189.4 | ± | 34.5 | 193.7 | ± | 35.4 | 0.144 | 191.5 | ± | 34.8 | 203.6 | ± | 33.6 | <0.001 |
| HDL-C (mg/dL) | 45.0 | ± | 10.5 | 43.9 | ± | 10.2 | 0.199 | 46.4 | ± | 9.9 | 44.3 | ± | 9.8 | 0.031 |
| LDL-C (mg/dL) | 112.7 | ± | 33.3 | 114.6 | ± | 35.7 | 0.499 | 117.0 | ± | 31.1 | 125.3 | ± | 31.2 | 0.006 |
| TG (mg/dL) | 158.7 | ± | 71.4 | 176.2 | ± | 78.2 | 0.008 | 140.7 | ± | 61.1 | 170.2 | ± | 67.8 | <0.001 |
| TG/HDL-C | 3.8 | ± | 2.2 | 4.4 | ± | 2.6 | 0.012 | 3.3 | ± | 1.9 | 4.1 | ± | 2.1 | <0.001 |

Data are expressed as mean ± standard deviation. Statistical differences between groups were compared with independent sample t-test.

Abbreviations: BMI=body mass index; WC= waist circumference; SBP= systolic blood pressure; DBP= diastolic blood pressure; FPG= fasting plasma glucose;2-hour PG=plasma glucose 2-hours post-glucose challenge; FPI=fasting insulin; 2-hour PI plasma insulin 2-hours post-glucose challenge; HOMA-IR= homeostatic model for insulin resistance; TC=total cholesterol; HDL-C= high-density lipoprotein cholesterol; LDL-C= low-density lipoprotein cholesterol; TG, triglyceride

Supplementary table4. Sex-stratified risk for incident diabetes by quartile of HOMA-IR among Participants with Normal Blood Pressure and prehypertension.

| **Normal BP** | **Number at risk** | **Diabetes cases** | **Unadjusted** | | | | | | **Model 1** | | | **Model 2** | | |
| --- | --- | --- | --- | --- | --- | --- | --- | --- | --- | --- | --- | --- | --- | --- |
|  |  |  | **HR (95% CI)** | | | | ***P* for**  **trend** | | **HR (95% CI)** | | ***P* for**  **Trend** | **HR (95% CI)** | | ***P* for**  **trend** |
| **Men** |  |  |  | |  | |  | |  |  |  |  |  |  |
| Quartile 1 | 443 | 49 | 1 | | (reference) | | 0.021 | | 1 | (reference) | 0.013 | 1 | (reference) | 0.100 |
| Quartile 2 | 444 | 53 | 1.05 | | (0.71-1.54) | |  |  | 1.06 | (0.72-1.56) |  | 1.04 | (0.70-1.53) |  |
| Quartile 3 | 444 | 54 | 1.08 | | (0.73-1.59) | |  |  | 1.10 | (0.75-1.62) |  | 1.02 | (0.69-1.51) |  |
| Quartile 4 | 443 | 73 | 1.53 | | (1.06-2.20)^*^ | |  |  | 1.58 | (1.10-2.28)^*^ |  | 1.39 | (0.95-2.02) |  |
| **Women** |  |  |  | |  | |  | |  |  |  |  |  |  |
| Quartile 1 | 541 | 47 | 1 | | (reference) | | 0.004 | | 1 | (reference) | 0.003 | 1 | (reference) | 0.041 |
| Quartile 2 | 537 | 40 | 0.86 | | (0.56-1.31) | |  |  | 0.91 | (0.59-1.38) |  | 0.86 | (0.56-1.32) |  |
| Quartile 3 | 539 | 49 | 1.03 | | (0.69-1.54) | |  |  | 1.07 | (0.72-1.60) |  | 0.96 | (0.64-1.45) |  |
| Quartile 4 | 539 | 77 | 1.60 | | (1.11-2.30)^*^ | |  |  | 1.66 | (1.15-2.38)^*^ |  | 1.41 | (0.97-2.04) |  |
| **Prehypertension** | | | | | | | | | | | | | | |
| **Men** |  |  | |  | |  | |  |  |  |  |  |  |  |
| Quartile 1 | 237 | 43 | | 1 | | (reference) | | 0.119 | 1 | (reference) | 0.066 | 1 | (reference) | 0.421 |
| Quartile 2 | 237 | 28 | | 0.63 | | (0.39–1.01) | |  | 0.63 | (0.39–1.02) |  | 0.62 | (0.38–1.00)^*^ |  |
| Quartile 3 | 238 | 47 | | 1.05 | | (0.69–1.58) | |  | 1.08 | (0.71–1.63) |  | 0.97 | (0.63–1.47) |  |
| Quartile 4 | 237 | 50 | | 1.21 | | (0.81–1.83) | |  | 1.29 | (0.86–1.95) |  | 1.04 | (0.67–1.60) |  |
| **Women** |  |  | |  | |  | |  |  |  |  |  |  |  |
| Quartile 1 | 204 | 31 | | 1 | | (reference) | | 0.700 | 1 | (reference) | 0.621 | 1 | (reference) | 0.805 |
| Quartile 2 | 205 | 33 | | 1.03 | | (0.63–1.68) | |  | 1.03 | (0.63–1.68) |  | 0.97 | (0.59–1.59) |  |
| Quartile 3 | 205 | 25 | | 0.75 | | (0.44 –1.26) | |  | 0.76 | (0.45–1.29) |  | 0.71 | (0.42–1.20) |  |
| Quartile 4 | 204 | 38 | | 1.20 | | (0.75–1.94) | |  | 1.23 | (0.76–1.98) |  | 1.02 | (0.63–1.68) |  |

Model 1: adjusted for age.

Model 2: adjusted for the variables in model 1 and body mass index, family history of diabetes (yes or no), education (less than high school, high school or equivalent, or college or above), alcohol use (current or non-current), and smoking status (current or non-current).

^*^*P* < 0.05, ^**^*P* < 0.001.

Supplementary table5 Sex-stratified risk for incident diabetes by quartile of HOMA-IR among Participants with Normal Blood Pressure and prehypertension.

| **Normal BP** | | **Number at risk** | | **Diabetes cases** | **Unadjusted** | | | | | | **Model 1** | | | | | | | **Model 2** | | | | | |
| --- | --- | --- | --- | --- | --- | --- | --- | --- | --- | --- | --- | --- | --- | --- | --- | --- | --- | --- | --- | --- | --- | --- | --- |
|  |  |  |  |  | **HR (95% CI)** | | | | ***P* for**  **trend** | | **HR (95% CI)** | | | | | ***P* for**  **Trend** | | **HR (95% CI)** | | | | ***P* for**  **trend** | |
| **Men** | |  | |  |  | |  | |  | |  | | |  | |  | |  | |  | |  | |
| Quartile 1 | | 443 | | 49 | 1 | | (reference) | | 0.021 | | 1 | | | (reference) | | 0.013 | | 1 | | (reference) | | 0.156 | |
| Quartile 2 | | 444 | | 53 | 1.05 | | (0.71-1.54) | |  |  | 1.06 | | | (0.72-1.56) | |  |  | 0.86 | | (0.58-1.27) | |  |  |
| Quartile 3 | | 444 | | 54 | 1.08 | | (0.73-1.59) | |  |  | 1.10 | | | (0.75-1.62) | |  |  | 0.80 | | (0.54-1.18) | |  |  |
| Quartile 4 | | 443 | | 73 | 1.53 | | (1.06-2.20)^*^ | |  |  | 1.58 | | | (1.10-2.28)^*^ | |  |  | 0.75 | | (0.50-1.12) | |  |  |
| **Women** | |  | |  |  | |  | |  | |  | | |  | |  | |  | |  | |  | |
| Quartile 1 | | 541 | | 47 | 1 | | (reference) | | 0.004 | | 1 | | | (reference) | | 0.003 | | 1 | | (reference) | | 0.656 | |
| Quartile 2 | | 537 | | 40 | 0.86 | | (0.56-1.31) | |  |  | 0.91 | | | (0.59-1.38) | |  |  | 0.75 | | (0.49-1.14) | |  |  |
| Quartile 3 | | 539 | | 49 | 1.03 | | (0.69-1.54) | |  |  | 1.07 | | | (0.72-1.60) | |  |  | 0.80 | | (0.53-1.21) | |  |  |
| Quartile 4 | | 539 | | 77 | 1.60 | | (1.11-2.30)^*^ | |  |  | 1.66 | | | (1.15-2.38)^*^ | |  |  | 1.02 | | (0.69-1.50) | |  |  |
| **Prehypertension** | | | | | | | | | | | | | | | | | | | | | | |  |
| **Men** |  | |  | | |  | |  | |  | |  |  | |  | |  | |  | |  | |  |
| Quartile 1 | 237 | | 43 | | | 1 | | (reference) | | 0.119 | | 1 | (reference) | | 0.066 | | 1 | | (reference) | | 0.478 | |  |
| Quartile 2 | 237 | | 28 | | | 0.63 | | (0.39–1.01) | |  |  | 0.63 | (0.39–1.02) | |  |  | 0.61 | | (0.38–0.99)^*^ | |  |  |  |
| Quartile 3 | 238 | | 47 | | | 1.05 | | (0.69–1.58) | |  |  | 1.08 | (0.71–1.63) | |  |  | 0.73 | | (0.48–1.12) | |  |  |  |
| Quartile 4 | 237 | | 50 | | | 1.21 | | (0.81–1.83) | |  |  | 1.29 | (0.86–1.95) | |  |  | 0.78 | | (0.51–1.20) | |  |  |  |
| **Women** |  | |  | | |  | |  | |  | |  |  | |  | |  | |  | |  | |  |
| Quartile 1 | 204 | | 31 | | | 1 | | (reference) | | 0.700 | | 1 | (reference) | | 0.621 | | 1 | | (reference) | | 0.155 | |  |
| Quartile 2 | 205 | | 33 | | | 1.03 | | (0.63–1.68) | |  |  | 1.03 | (0.63–1.68) | |  |  | 0.90 | | (0.55–1.49) | |  |  |  |
| Quartile 3 | 205 | | 25 | | | 0.75 | | (0.44 –1.26) | |  |  | 0.76 | (0.45–1.29) | |  |  | 0.60 | | (0.35–1.03) | |  |  |  |
| Quartile 4 | 204 | | 38 | | | 1.20 | | (0.75–1.94) | |  |  | 1.23 | (0.76–1.98) | |  |  | 0.76 | | (0.46–1.27) | |  |  |  |

Model 1: adjusted for age.

Model 2: adjusted for the variables in model 1 and fasting plasma glucose, body mass index, family history of diabetes (yes or no), education (less than high school, high school or equivalent, or college or above), alcohol use (current or non-current), and smoking status (current or non-current).

^*^*P* < 0.05, ^**^*P* < 0.001.
